# Angiotensin-Converting Enzyme 2 Expression and Severity of SARS-CoV-2 Infection

**DOI:** 10.3390/microorganisms11030612

**Published:** 2023-02-28

**Authors:** Sarah Alabsi, Atharva Dhole, Sameh Hozayen, Scott A. Chapman

**Affiliations:** 1 University of Minnesota School of Medicine, 420 Delaware St. SE, Minneapolis, MN 55455, USA; 2 Department of Internal Medicine, University of Minnesota, 420 Delaware St. SE, Minneapolis, MN 55455, USA; 3 Hospital Medicine Division, Department of Medicine, University of Minnesota, Mayo Building 420 Delaware St. SE, 6 floor, D650, Minneapolis, MN 55455, USA; 4 Department of Experimental and Clinical Pharmacology, University of Minnesota College of Pharmacy, 7-115E Weaver Densford Hall, 308 Harvard Street SE, Minneapolis, MN 55455, USA

**Keywords:** angiotensin-converting enzyme 2, SARS-CoV-2, COVID-19

## Abstract

Angiotensin-converting enzyme 2 (ACE2), first discovered in 2000, serves as an important counterregulatory enzyme to the angiotensin II-mediated vasoconstrictive, pro-inflammatory, and pro-fibrotic actions of the renin–angiotensin system (RAS). Conversion of angiotensin II to the peptide angiotensin 1–7 (ANG 1–7) exerts protective vasodilatory, anti-inflammatory, and anti-fibrotic actions through interaction with the MasR receptor. There are many important considerations when noting the role of ACE2 in the pathogenesis and sequelae of COVID-19 infection. ACE2, in the role of COVID-19 infection, was recognized early in 2020 at the beginning of the pandemic as a cell membrane-bound and soluble binding site for the viral spike protein facilitating entering into tissue cells expressing ACE2, such as the lungs, heart, gut, and kidneys. Mechanisms exist that alter the magnitude of circulating and membrane-bound ACE2 (e.g., SARS-CoV-2 infection, viral variants, patient characteristics, chronic disease states, and the degree of cell surface expression of ACE2) and the influence these mechanisms have on the severity of disease and associated complications (e.g., respiratory failure, systemic inflammatory response syndrome, acute myocarditis, acute kidney injury). Several medications alter the ACE2 receptor expression, but whether these medications can influence the course of the disease and improve outcomes is unclear. In this review, we will discuss what is known about the interrelation of SARS-CoV-2, ACE2 and the factors that may contribute to the variability of its expression and potential contributors to the severity of COVID-19 infection.

## 1. Introduction

Soon after the SARS-CoV-2 outbreak in Wuhan, China, in late 2019, it was discovered that SARS-CoV-2 (like SARS-CoV) gains entry into cells via interaction between the receptor-binding domain (RBD) of the S1 subunit of the viral spike glycoprotein and the peptidase domain of angiotensin-converting enzyme 2 (ACE2) [1]. The S2 subunit then uses a hydrophobic fusion peptide domain to mediate the interaction between host and viral membranes [2]. SARS-CoV-2 has demonstrated much higher infectivity than SARS-CoV, evidenced by its 10–20 times greater binding affinity to ACE2 [3]. Once bound to ACE2, the viral spike glycoprotein on the SARS-CoV-2 cell surface becomes activated by TM protease serine 2 (TMPRSS2) which promotes viral entry into cells where mRNA is then released into the cytoplasm, initiating infection [4].

**Figure 1:**
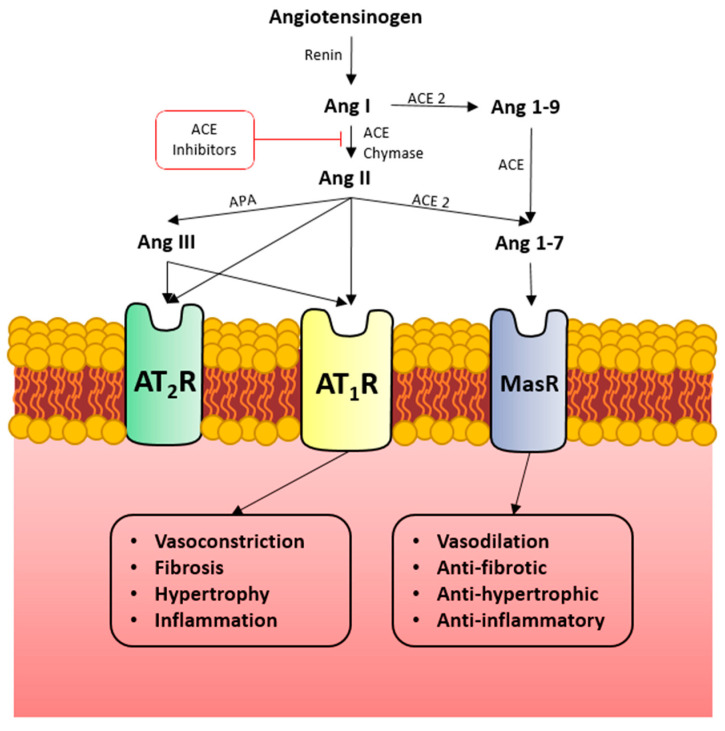
Angiotensin-converting enzyme 2 (ACE2) functions by converting Angiotensin 1 (Ang I) and Angiotensin 2 (Ang II) to Angiotensin 1–9 (Ang 1–9) and Angiotensin 1–7 (Ang 1–7), respectively. Ang 1–9 can further be converted to Ang 1–7; Ang 1–7 acts at the MAS1 oncogene (MasR). Ang II can act directly at the Angiotensin Receptor Type 1 (AT1R) or be converted into Angiotensin III (ANG III) by aminopeptidase (APA), which can act at both AT1R or Angiotensin Receptor Type 2 (AT2R). AT1R functions to increase vasoconstriction, fibrosis, hypertrophy, and inflammation, whereas MasR opposes each of these effects. The increased presence of ACE2 catalyzes the shift from Ang II-mediated processes toward those mediated by Ang 1–7, thus influencing the balance in these opposing forces.

Discovered in 2000 by two independent research groups, ACE2 is a 42% homolog to angiotensin-converting enzyme (ACE). It was identified as a counter-regulatory enzyme to the renin–angiotensin–aldosterone system (RAAS) by hydrolyzing the peptide angiotensin II (ANG II) to angiotensin 1–7 (ANG1–7) and angiotensin I to angiotensin 1–9. Angiotensin 1–9 is further converted to ANG1–7 [5,6]. ANG1–7 is a vasorelaxant and antifibrotic peptide that binds to the Mas receptor (MasR). The Ang II-Ang-(1–7)-MasR axis has been a key player in various physiologic and pathophysiologic processes in the body [7] (Figure 1).

Circulating ACE2 can occur when ADAM17 (a disintegrin and metalloproteinase 17) cleaves ACE2 from the cell membrane. Initially, ACE2 gene expression was identified in the heart, kidney, and testes [5,6]. Today, ACE2 is known to be widely distributed in many tissues, including the lungs, gut, and liver [8]. These organ systems have been identified as targets of COVID-19 disease and associated with the sequelae of infection [9]. In COVID-19 infection, the virus spike glycoprotein binding to ACE2 blocks the activity of ACE2, leaving ANG II activity relatively unopposed. The resulting imbalance, favoring increased ACE/ANG II/ANG 1 receptor axis activity, manifests in greater vasoconstrictive, pro-fibrotic, thrombotic, inflammatory, and oxidative forces [10]. Additionally, the relatively diminished opposition from the anti-inflammatory ANG 1–7 precipitates multi-organ damage [11,12]. Several factors have been associated with altering the expression of ACE2, raising concern for severe COVID-19 infection and poor outcomes. These factors include patient characteristics and ACE2 polymorphisms, comorbidities, and medication use.

In this review, we will explore the implications of ACE2 expression differences, primarily whether or not these differences correlate to differential infection susceptibility, disease severity, or post-acute sequelae of COVID-19 infection (PASC).

## 2. Methods

A comprehensive search of the literature using electronic databases (Medline, PubMed, Web of Science, and Google Scholar) to identify publications on ACE2 in disease and COVID-19 was performed to select articles for inclusion. The range of search publication dates ranges from 2000–2022. This date range was selected based on the year of ACE2 discovery to the present. All authors contributed to the search and review of the literature using search terms related to the focus of the manuscript. The literature review yielded 132 publications. Review articles, commentaries, and experimental studies were not excluded in favor of a broad overview of the recent literature.

## 3. Demographic Characteristics

Angiotensin-converting Enzyme-2 is a peptide with 42% homology to ACE. It is located on band p22.2 on the short arm of the X chromosome. The ACE2 protein contains a terminal signal peptide region, metal shuttle peptidase activation site, and transmembrane region [13]. Its presence on the X chromosome lends itself to questions on sex-based differences, as the phenomenon of X inactivation in females can cause differential expression of certain genes when compared to males [11]. Specifically, in this case, ACE2 is present on a portion of the X chromosome known for escaping inactivation, leading to a potential over-expression of ACE2 in female cells [11].

Although the likelihood of COVID-19 infection is not significantly different between the male and female sex, the severity of the disease seems to be higher in males [14]. In 2020 the mortality rate was found to be 77% higher in males than in females [15].

Some comparisons of ACE2 expression in humans based on sex have not found consistent significant differences [16]. A substantial portion of the research looking into sex-based differences in ACE2 has concentrated on the presence of single nucleotide polymorphisms (SNPs) between the sexes and whether these SNPs have been implicated in differential disease severity. For example, a case-control study found that five ACE2 polymorphisms were significantly associated with essential hypertension in Chinese Han females [17]. Furthermore, this study also showed that ACE2 activity level was positively correlated with estrogen levels in females with essential hypertension.

Hormonal factors may also lead to ACE2 overexpression in females [18]. Estrogen has been observed to play a protective role against SARS by directly suppressing the replication of SARS-CoV. It has also been known to upregulate ACE2 expression, leading to a beneficial decrease in the ACE/ACE2 ratio [19]. Animal models have shown less evidence of a significant difference in ACE2 expression. Male and female rats of the same age did not show a significant difference in pulmonary ACE2 expression though the age-related decline in ACE2 expression was more evident in male than female rats [20,21].

Age is another factor that has been known to cause differential susceptibility and severity of COVID-19 infection. The knowledge that COVID-19 infection disproportionately affects older individuals became widely known soon after the discovery of the virus [22,23]. In addition to the devastating effects of social isolation due to COVID-19 in this age population, the virus itself has a dangerous proclivity for severe infection and mortality in the elderly [24]. One early study showed that patients 60 years and older were almost 20 times more likely to die from COVID-19 infection [25]. In the United States, 80% of deaths due to COVID-19 infection occurred in patients 65 and older [26]. There are many likely reasons for this disparity, including a higher prevalence of chronic disease, polypharmacy, immunocompromisation, and group housing [26].

Research findings on ACE2 expression changes with aging have not been consistent. Meng-Yuan Li et al. investigated these effects across a wide variety of human histology, comparing ACE2 expression in 31 human tissue types in persons aged ≤49 years and aged >49 years. They found no significant differential expression between the age groups across all tissue types [16]. In another study, no significant age-based change in expression was detected in multiple tissues, although a nonsignificant trend of decreasing ACE2 expression with increased age was observed and more evident in males than females [27].

Some tissues tend to have more data to support differential ACE2 expression based on age, particularly in rats. In the lungs, research has shown that ACE2 expression tends to decrease to a significant level with increased age [20]. This experiment, performed in rats at three months, 12 months, and 24 months of age, found that the inverse relationship was more dramatic in males. Between the rats aged three months and 24 months, a 78% and 67% decrease in ACE2 expression was noted in males and females, respectively [20]. The expression also increases with age in the lower nasal [28] and bronchial [29] epithelial cells. These findings in the nasal epithelium were lowest in young children <10 years and highest in adults ≥25 years. The positive correlation between age and ACE2 expression in this tissue could partially explain disparities in infectivity based on age [28]. Furthermore, in both children and adults, ACE2 nasal tissue levels were significantly higher than in bronchial epithelial tissues [29]. However, in adults, ACE2 expression in the nasal epithelium was far greater than expression in the blood or saliva, allowing the researchers to hypothesize on a mechanism for why rates of infectivity are lower in younger populations [29]. The increase of ACE2 expression with age was found to be comparatively more evident in ventilated patients than in non-ventilated patients with ARDS due to SARS-CoV-2 [30]. This study showed that ACE2 RNA and protein expression in type II pneumocytes was only significantly increased with age in samples from individuals on mechanical ventilation in the setting of alveolar damage. Non-ventilated individuals did not demonstrate these changes in type II pneumocytes and experienced no such age-related increase in expression [30]. Silva et al. demonstrated similar findings in type II pneumocytes and consequently identified that angiotensin II levels in children were higher than those in adults [31]. Ang-(1–7)/Ang II ratios served as surrogate markers for ACE2 activity in this study and were higher in adults [31].

## 4. Disease States

### 4.1. Cardiovascular Disease

Early in the start of the SARS-CoV-2 pandemic, it became evident that those who had the cardiovascular disease were at increased risk for severe infection and morbidity [32]. Furthermore, infection with SARS-CoV-2 has been linked to cardiovascular sequelae, including myocardial injury and dysfunction [32]. In the cardiovascular system, cardiomyocytes, epicardial adipose tissue, cardiac fibroblasts, vascular smooth muscle, and endothelial cells express membrane-bound ACE2 [8]. Single-cell RNA-sequence studies have shown that as many as 7.5% of myocardial cells had ACE2 expression. The high level of expression of ACE2 on myocardial cells elevates the risk for SARS-CoV-2 entry and subsequent ACE2 downregulation resulting in cardiac sequelae of COVID-19 infection [33].

Early after its discovery, the role of ACE2 as an essential regulator of cardiovascular function was demonstrated. In a landmark study, ACE2 knock-out mice demonstrated impaired cardiac contractility, with male mice and older mice showing more significant impairments [34]. Increased ANG II levels were also observed in the ACE2 knock-out mice. These cardiac phenotypes were not present when ACE and ACE2 were both genetically ablated in mice. Furthermore, coronary vasoconstriction due to the loss of the receptor led to decreased myocardial perfusion and upregulated markers of hypoxia [34].

Given the potentially dangerous cardiac effects of poor ACE2 expression, there were many research studies examining the predictive value of ACE2 expression on CVD years before the first SARS-CoV-2 case was even discovered. In Zhang et al., a case-control study showed that serum ACE2 enzymatic activity could predict the development of cardiac dysfunction in patients with essential hypertension [17]. Similar to ACE2 levels that affect the likelihood of developing cardiovascular symptoms, cardiovascular injury has also been shown to alter ACE2 expression levels.

In the United States alone, it is estimated that more than 800,000 acute myocardial infarctions (AMIs) occur each year [35]. In the wake of a MI, transient activations of RAAS result in plasma ACE and ANG II elevation, the latter of which can lead to devastating cardiac remodeling [36]. This is also known as the underlying pathology behind the mortality-reducing efficacy of ACE inhibitors after MI [37,38,39]. In the hours to months following a MI, many changes happen at the cellular level as the heart goes through a series of steps to remodel damaged tissue. Three days following MI induction in rats via ligation of the left coronary artery, an increase in myocardial ACE2 expression was observed in two areas: the zone of infarct and the border zone, a transition area between the infarcted scar tissue and the normal myocardium [40]. Increased ACE2 expression was also noted in unaffected myocardial tissues after 28 days [40]. Further immunohistochemistry showed that elevated ACE2 was being expressed in mononuclear cells, known to remove debris in the facilitation of tissue repair [41]. As the vasodilating ANG 1–7 is a product of ACE2 activity, it is likely that the ACE2-angiotensin-(1–7)-MasR axis functions to counter-regulate the effects of RAAS [40,42]. This, in turn, would be cardioprotective. In support of this claim, an experiment providing ACE2 inhibitors to rats post-MI resulted in increased infarct size, likely due to lower coronary perfusion, as well as a reduction in left ventricular contractility [43]. While only MI has been mentioned here, heart failure is a common sequela of MI, with 30–40% of MI patients developing heart failure at some point following diagnosis [44]. Due to the wealth of research showing elevated expression of the ACE2 receptor in these CVD states, researchers were quick to suspect heart failure patients were a higher-risk population at the onset of the SARS-CoV-2 pandemic [45].

While heart failure may result in increased expression of the ACE2 receptor, it seems that infection of cardiac myocytes with SARS-CoV-2 leads to downregulation of its expression [46]. Consequently, this leads to the upregulation of RAAS and ANG II in COVID-19 patients, potentially exasperating the cardiovascular system of patients with prior cardiac, hypertensive, and/or diabetic history. These conditions, as well as old age, have been associated with variable rates of ACE2 deficiency, providing yet another mechanism for predisposition to severe COVID-19 infection [47].

### 4.2. Hypertension

In addition to its notable counterregulatory role to ANG II as a vasodilator, the creation of ANG 1–7 results in a decrease in water retention and salt intake in the kidneys [48]. Thus, it follows that several studies have shown that ACE2 deficiency—whether by deletion or inhibition—may be a contributor to hypertension. The receptor enzyme can no longer exert its protective regulation of ANG II, resulting in uncontrolled vasoconstriction [49,50]. Experiments in rats have shown decreased ACE2 mRNA, and protein expression in both cardiac myocytes and nephrocytes has been associated with primary hypertension that is reversed with the introduction of ACE2 [51,52]. In fact, knowledge of this lack of control on the RAAS led to concern early in the COVID-19 pandemic regarding the use of angiotensin receptor blockers (ARBs) and ACE inhibitors in patients infected with the virus. A great number of observational studies and trials were subsequently conducted on hospitalized COVID-19-infected patients with preexisting hypertension to examine whether inpatient use of these medications was leading to adverse outcomes [53,54,55]. A 2020 meta-analysis of nearly 10,000 hypertensive subjects across 10 studies revealed that the use of either or both of these medications resulted in similar rates of severe or lethal COVID-19 infection [55]. Medical guidance has since moved away from the early recommendations advising the discontinuation of ACEi and ARBs in COVID-19 infected individuals, and now recommend continuing these therapies if clinically indicated during COVID-19 infection.

Given the extensive research demonstrating the protective role of ACE2- ANG (1–7)– MasR axis on blood pressure lowering effects, it has been hypothesized that SARS-CoV-2 infection interferes with ACE2′s ability to function optimally in this axis [56]. Consequently, hypertension has remained a risk factor in clinical practice for severe COVID-19 infection. This is likely an appropriate response as a meta-analysis synthesizing results from early COVID-19 studies showed hypertensive patients had 2.27- and 3.48-fold higher risk of severity and fatality due to COVID-19 infection, respectively [56]. ACE2 downregulation or inactivity poses a serious threat to human health as overactivation of the RAAS system is often a contributor to multi-organ failure [57].

### 4.3. Diabetes

Patients with diabetes mellitus face increased incidence and severity of COVID-19 infection [58], with one meta-analysis finding that diabetic patients have a two-fold increase in mortality [59]. Within the kidney, ACE2 is expressed primarily on proximal tubule cells and the podocytes of glomeruli [60]. ACE2 is also expressed in adipose tissue and the pancreas, particularly islet cells and exocrine glands crucial for blood sugar homeostasis [8].

As seen in hypertension, individuals with diabetic nephropathy have been shown to have a decrease in ACE2 expression in their kidneys [48]. This finding was not reproducible to a significant level in other causes of nephropathy, including focal segmental glomerulosclerosis and chronic allograft nephropathy, suggesting a diabetes-specific mechanism. In one study examining renal biopsies of diabetic patients, ACE2 mRNA levels were reduced by more than half in comparison to healthy controls [61].

A large phenom-wide Mendelian randomization study conducted in 2020 examined the relationship between ACE2 lung expression (and consequently, susceptibility to SARS-CoV-2 infection) and diabetes-related traits [62]. In this way, ACE2 expression was used as the outcome measure while various disease states and traits, including diabetes, were analyzed as exposures. Type 2 diabetes mellitus was found to be causally linked to a rise in global ACE2 expression. Similar findings were seen in type 1 diabetic individuals. They concluded that the increase in the expression of ACE2 would likely influence susceptibility to SARS-CoV-2 infection or the severity of infection [62].

Perhaps unsurprisingly, those with uncontrolled diabetes tend to fare worse when infected with SARS-CoV-2. This includes increased rates of severe infection, mortality, and, potentially, reinfection [63,64]. Additionally, an imbalance in the ANG II/ANG-(1–7) ratio was observed in type 2 diabetes patients, exacerbating hyperglycemia and leading to a higher propensity for vascular dysfunction when compared to patients without a ratio imbalance [65]. Thus, an important part of COVID-19 treatment in these patients needs to be proper control of their blood sugars.

### 4.4. Kidney Disease

Kidney injury is a frequent disease encountered with COVID-19 infection. A systematic review and meta-analysis of over 30,000 patients hospitalized with COVID-19 infection demonstrated that the prevalence of acute kidney injury (AKI) was 28% and as high as 46% in ICU patients [66]. It has been suggested that the mechanism by which AKI is induced in COVID-19 infection is by viral antigen immune complex deposition or cytokine-mediated local injury, both of which can induce hypoxia, shock, or rhabdomyolysis [67]. Another mechanism by which COVID-19 is able to affect the kidney and induce AKI is through direct kidney parenchymal infection. This is evidenced by the presence of viral particles in podocytes, resulting in their detachment from the glomerular basement membrane [68].

Soon after it was postulated that the entry of SARS-CoV was facilitated by ACE2, studies identified its presence in weak-to-abundant densities in the kidney, depending on location [69]. One post-mortem renal histopathological analysis in COVID-19 patients who died from respiratory failure found an increased expression of ACE2 in the kidneys of COVID-19 patients compared to healthy controls from archival biopsy reports [68].

Furthermore, in COVID-19 patients, chronic kidney disease (CKD) in stages 3–5 was found to be a significant independent variable associated with mortality [70]. This relationship extended even within varying severities of CKD; compared to patients with CKD stages 1–2, those in stages 3–5 were significantly more likely to require ICU hospitalization, develop AKI, have longer hospitalization stays, and/or have higher inflammatory markers (CRP, ferritin, D-dimer) [70,71]. Given the complexity of the COVID-19 inflammatory cascade and variability of infection, the etiology of renal tissue damage in the context of CKD (possibly more in 3–5 than in 1–2) is equally variable. This was further corroborated in a rat model with advanced CKD, in which immunohistochemistry demonstrated a strong expression of ACE2 in damaged proximal tubular cells which corresponded with lesions of COVID-19-induced AKI [72]. Consequently, this leaves this cohort of patients susceptible to entering a vicious cycle of an increased likelihood of developing COVID-19-induced AKI [73] and a subsequent accelerated progression of their pre-existing CKD and mortality risk [74].

One study, which aimed to characterize the kidney ACE2 mRNA expression between healthy living donors and subjects with CKD, was able to identify several distinctions in this expression, specifically between the tubulointerstitial and glomerular compartments. While expression was similar between the two groups in the tubulointerstitial compartment, albeit with vastly greater variance in the CKD cohort, there was a statistically significant reduced expression for CKD subjects in the glomerular compartment [60].

Another cohort of patients in whom COVID-19 was particularly destructive includes kidney transplant recipients (KTRs). A meta-analysis of over 70 studies revealed that 25% of KTRs required intensive care treatment, and 23% died, all with substantial heterogeneity [75]. Additional outcomes included rejection and graft failure (0.16 and 0.86%, respectively), dialysis (6%), and AKI (50%). The median duration of hospitalization ranged from 4 to 36 days [75]. Another meta-analysis found that 45% of KTRs developed Acute Respiratory Distress Syndrome (ARDS), and 23% required intubation [76]. The rates of comorbidities in these patients clearly outpaced those of patients with native kidneys. It was this dramatic and morbid presentation in this vulnerable population that prompted Cahova et al. to evaluate the effect of RAAS inhibitors on ACE2 expression in KTRs (n = 48). They found that ACE2 expression was not significantly affected by immunosuppressive (T cell-depletive) therapies or by the use of RAAS inhibitors [77].

### 4.5. Pulmonary Diseases

COVID-19, conventionally regarded as a respiratory illness, has manifested in pulmonary pathology of varying severity. Pulmonary COVID-19 is similarly mediated via ACE2 receptors which have been found in greatest abundance in type 2 pneumocytes and macrophages [47]. Other common areas of ACE2 expression in the lungs include the lung fibroblasts and bronchial and tracheal epithelial cells [69]. Acute respiratory distress syndrome (ARDS) is considered a frequent and prominent complication of inpatient COVID-19 infection [78]. The role of ACE2 and its polymorphisms was well explored even in the early 2000s, where it was documented that higher expressions translated to greater severity of ARDS and outcomes [79].

ACE2, conversely, was shown to have a protective effect on ARDS. Likewise, ACE2-deficient samples performed much worse and were susceptible to many morbidities, including hypoxia, sepsis, and overall worsened respiratory function [80]. A study conducted by Yamaguchi et al. elucidated and provided greater breadth to this statement. Researchers found that the SARS-CoV-2 spike protein, with and without the addition of acid aspiration, resulted in the downregulation of ACE2 in the lungs of hamsters and mice. This was followed by pulmonary edema, neutrophil infiltration, destruction of alveolar architecture, and ultimately, the development of severe ARDS [81]. Winkler et al. reinforced these findings when they devised a mice model in which human ACE2 (hACE2) knock-in mice were intranasally inoculated with the COVID-19 virus. They subsequently found that there was substantial viral replication in the respiratory tracts but limited pathology. Their histopathological findings showed preserved airway structure and significantly reduced proinflammatory cytokine and chemokine burden [82]. The mechanism by which this happens is due to the unopposed activation of the ACE-bradykinin pathways, which ACE2 inhibits. This results in angioedema and vascular leakage, thereby precipitating ARDS [83]. However, it is worth noting that ACE2 expression changes alone do not provide an all-encompassing predictor of COVID-19 severity; pulmonary fibrosis, as sometimes seen in severe COVID-19 infection, has been shown to result due to inhibition of ACE2 [84,85].

Chronic obstructive pulmonary disease (COPD) has been associated with worsened outcomes and increased severe COVID-19 infections due to its increased expression of ACE2 [86]. Leung et al. were the first to use resected lung tissue specimens and bronchial brushings to quantify ACE2 expression in active smokers with COPD. They further collected samples from nonsmoker controls and healthy active smokers. Their results included significantly increased ACE2 expression in lung epithelial cells in COPD versus non-COPD subjects and in smokers versus non-smokers, providing a possible explanation for why COPD was associated with worsened COVID-19 outcomes [87]. The consequence of this is an association between COPD and COVID-19 mortality with an odds ratio of 2.1, hospitalization OR of 1.27, and intensive respiratory support OR of 1.47 [88,89].

While it is well known that smoking is the leading cause of COPD [90], recent data has also shown that chronic tobacco smoke exposure is associated with a dose-dependent increase in ACE2 expression within both human and rodent lungs [91]. This is hypothesized to be due to the metaplasia of ACE2-expressing secretory cells as a consequence of smoke inhalation. These expression differences are particularly noticeable in the lower airways [87]. Nicotine-containing vapor from e-cigarettes has also been directly linked to significantly increased ACE2 mRNA and protein expression in the lungs of male mice [92]. The reason for this sex-specific finding is still unknown.

Active asthma, similarly, carried with it an increased odds of hospitalization, ICU admission, and intensive respiratory support (IOR 1.47–1.66) as one population-based study (n = 61,338) on COVID-19 severity discovered [89]. Despite the increased severity of morbidity during infection, patients with active and inactive asthma, regardless of whether or not the patient was receiving medication for asthma control, experienced no significant increase in mortality rates [89]. ACE2 expression in asthma, however, is variable depending on the characterization of asthma itself. One study found that for atopic asthma, there is actually a low ACE2 expression, whereas, for nonatopic asthma, there was no association with low ACE2. The author extrapolated this to suggest that atopic, and not nonatopic, asthma correlated with reduced COVID-19 severity [93].

Excessive alcohol consumption has been associated by some with worse severity and poorer outcomes of COVID-19 [94], and high alcohol consumption is considered a risk factor for ARDS [95]. A study of 171 patients infected with SARS-CoV-2 who self-reported alcohol consumption reported that a higher amount of alcohol consumption was associated with ARDS in univariate and multivariate analyses [96]. An experiment in a mouse model involving chronic alcohol exposure found significant upregulation of ACE2 expression in the lungs of alcohol-exposed animals compared to control mice [97]. Another recent experiment exploring ACE2 expression in SARS-CoV-2 infection and alcohol exposure was conducted in an ARDS model involving human ACE2 transgenic mice to investigate the association between ethanol exposure and pulmonary ACE2 expression. Mice were fed either a control diet or an ethanol diet and administered the subunit of SARS-CoV-2 spike protein (S1SP) intratracheally. At 72 h after S1SP was administered, compared to control mice, the ethanol diet mice had higher expression of ACE2 as well as evidence of higher activation of proinflammatory biomarkers [98].

## 5. Medications

### 5.1. Angiotensin-Converting Enzyme Inhibitors and Angiotensin Receptor Blockers

Angiotensin-converting enzyme inhibitors and angiotensin receptor blocker (ARBs) agents have been explored for their effects on the expression of ACE2. Angiotensin-converting enzyme inhibitors block the production of ANG II through its conversion from ANG I. Angiotensin II receptor antagonists directly inhibit the angiotensin type 1 receptor.

The relationship between the use of these medications and disease severity and/or patient outcomes was uncertain early in the COVID-19 pandemic. A challenge arose in balancing the risk of worsening health outcomes by discontinuing these agents with the risk of possibly exacerbating COVID-19 infection due to the still unclear relationship of ACE2 expression. These agents have been reported to induce the upregulation of ACE2 in animal models [99]. In one experiment, Lewis rats were administered lisinopril and losartan to examine the response of cardiac ACE2 expression. Rats administered lisinopril were found to have an increase in cardiac ACE2 mRNA expression only, while rats administered losartan were found to have both an increase in cardiac mRNA expression and increased ACE2 expression [99]. Another animal study administered the ARBs losartan or olmesartan to the Lewis rat model of myocardial infarction. The rats who received ARBs were found to have a three-fold increase in ACE2 mRNA expression compared to animals who received a placebo [100]. Cardiac ACE2 mRNA expression was also increased by candesartan in a hypertension model in salt-sensitive Dahl rats [101].

Concern regarding ACE inhibitors and ARB exposure leading to greater expression of ACE2 is particularly strong given that patients on these medications often have characteristics and comorbidities such as CVD, hypertension, and diabetes that each may make them more susceptible to SARS-CoV-2, as previously described earlier in this review [102]. This concern was addressed in a comprehensive review of the RAAS in COVID-19 [103]. However, in this review, the authors note that there is inconclusive evidence linking ACE inhibitors and ARBs to poorer outcomes in COVID-19. Additionally, withdrawal of these agents from patients with clear indications, good tolerance, and who have no other reason for their discontinuation would risk putting these patients in harm. A systematic review and meta-analysis of 10 studies evaluating patients who were SARS-CoV-2 positive and were or were not taking ACEI or ARBs further found no increased risk of mortality or severity of clinical manifestations [104]. Another study evaluating the effects of ACE inhibitors, ARBs, and mineralocorticoid receptor antagonists (MRAs) in heart failure patients on soluble plasma concentrations of ACE2 found that ACE inhibitor or ARB use was not a strong predictor of elevated circulating ACE2 levels. This study did find that the strongest predictor of elevated circulating plasma ACE2 levels was in men. The authors concluded from these findings that these agents do not increase the risk for severe COVID-19 infection [105]. Organization statements on ACE inhibitors or ARB use have since recommended the continuation of these agents beyond the clinical care needs of the patient independent of COVID infection [106].

### 5.2. Mineralocorticoid Receptor Antagonists

Aldosterone serves an important role in blood pressure regulation through its promotion of sodium and water retention at the distal tubule of the kidney, while angiotensin II promotes the production of aldosterone. Studies in human and animal models have reported that aldosterone reduces ACE2 activity, and mineralocorticoid receptor antagonists increase ACE2 activity. In a neonatal rat cardiac myocyte model, aldosterone reduced ACE2 mRNA levels [107]. Furthermore, both the aldosterone receptor antagonists spironolactone and eplerenone have been reported to increase ACE2 activity and mRNA ACE2 expression. In 10 male heart failure patients, ACE2 activity and mRNA expression in monocyte-derived macrophages were increased by 300% and 654%, respectively, from baseline after one month of spironolactone. This same study also measured the effect of eplerenone on cardiac ACE2 in mice and found that, compared to controls, treated mice had a 2.54-fold higher ACE2 activity in monocyte-derived macrophages and a 99% increase in ACE2 activity in hearts [108]. Aldosterone decreased ACE2 activity in the mouse’s peritoneal macrophage cultures, and the addition of eplerenone reversed these effects [108]. Another study evaluating the glomerular effects of aldosterone in rats associated glomerular damage by aldosterone to downregulation of ACE2 gene expression. Animals receiving co-administration of eplerenone reduced these effects [109]. Hypokalemia associated with SARS-CoV-2 infected patients suggested disruption of the RAAS, possibly signaling hyperaldosteronism as a primary cause of hypokalemia [110]. Another report of 188 patients hospitalized with COVID-19 infection showed that low aldosterone:renin ratios may be predictive of COVID-19 severity of disease [111]. Furthermore, some authors have suggested that mineralocorticoid receptor antagonists may be protective against SARS-CoV-2 due to their effects on ACE2 and RAAS as well as their anti-androgen, anti-fibrotic, and anti-hyperinflammatory actions [112].

### 5.3. HMG CoA Reductase Inhibitors

ACE2 has also been studied for its role in preventing atherosclerosis. It has been hypothesized that HMG CoA reductase inhibitors (statins) can have a role in association with ACE2 on the development of atherosclerosis and its prevention. An experimental model involving high-cholesterol-diet (HCD) rabbits reported reduced expression of ACE2 in HCD-fed control rabbits and upregulation of ACE2 in HCD-fed rabbits treated with atorvastatin [113]. In a diabetic rat model, the addition of fluvastatin (with insulin) improved cardiac ACE2 expression and decreased myocardial fibrosis when compared to rats treated with insulin alone [114]. Similar findings were reported in a rat model of diabetic cardiomyopathy, where the addition of atorvastatin improved ACE2 expression and normalized ACE/ACE2 ratio compared to control animals who did not receive atorvastatin [115]. Measurements of ACE2 mRNA expression were increased in rats who received rosuvastatin compared to controls after vascular balloon injury [116].

One cohort study found a slight but statistically significant negative association between the use of statins and COVID-19 mortality. In this study, participants were included if they had begun using statins before the beginning of the pandemic [117]. Another cohort study retrospectively examined the use of statins as COVID-19 adjuvant treatment within the ICU, finding they were able to reduce in-hospital and 30-day mortality to a significant degree [118].

### 5.4. Metformin

Metformin exerts protective effects on the cardiovascular and pulmonary systems. These protective effects may be associated with the activation of AMP-activated protein kinase (AMPK), a multifunction enzyme that serves to maintain energy homeostasis through reduced cellular ATP:ADP and ATP:AMP ratios [119]. Through the phosphorylation of ser-680 on ACE2 by AMPK, the phosphorylated ACE2 undergoes conformational changes that provide increased stability and a prolonged half-life. The increased stability of phosphorylated ACE2 leads to an increase in ANG 1–7 as well as endothelial nitric oxide synthase-derived nitric oxide [120]. In a mouse model of endothelial homeostasis and pulmonary hypertension, ACE2 S680D gain of function knock-in mice was found to be resistant to developing pulmonary hypertension when compared to wide-type littermates. Meanwhile, ACE2 knock-out mice experienced an exacerbation of pulmonary hypertension [108]. Others hypothesize that metformin increases circulating ACE2 and, in doing so, decreases morbidity and mortality associated with COVID-19 infection. Additionally, the conformation changes associated with ACE2 phosphorylation may reduce the binding affinity of the SARS-CoV-2 spike protein [121].

Some retrospective clinical investigations of metformin use during COVID-19 infection [122,123,124], as well as in one systematic review and meta-analysis [125], found an associated mortality benefit. However, another prospective randomized trial in COVID-19 infection found no mortality outcome benefit with metformin as the primary endpoint [126]. This same study found that those taking metformin were 42% less likely to have COVID-19 infection that resulted in emergency room visits, hospitalization, or death [114].

### 5.5. Glucagon-like Peptide-1 Receptor Agonists

The glucagon-like peptide-1 receptor agonists (GLP-1 RAs) may be protective of the lungs in SARS-Co-V2 infection through their ability to increase ACE2 expression [127]. The GLP-1 RAs act to directly stimulate the GLP-1 receptor, thereby increasing insulin secretion and decreasing glucagon release to maintain glucose homeostasis. They also work by exerting anti-inflammatory effects through the inhibition of cytokine release [128]. The GLP-1 RA liraglutide improved ACE2 expression and ANG1–7 in the lungs of both experimental diabetic and control rats in a model of lung hypoplasia [129]. Additionally, GLP1 receptor agonists have been found to induce the synthesis of lung surfactant proteins which are protective in bacterial and viral infections [130].

### 5.6. Dipeptidyl Peptidase-4 Inhibitors

Dipeptidyl peptidase-4 (DPP4) is expressed in many tissues and serves several functions, including the cleavage and inactivation of GLP-1 [131]. DPP4 inhibitors prevent the breakdown of GLP-1, thus exerting anti-inflammatory effects by reducing cytokine production. It has been theorized that similar to MERS-CoV, SARS-CoV-2 spike protein could also bind to DPP4. In a nephrectomy rat model, sitagliptin was found to reverse the effects of reduced ACE2 expression. Compared to controls, animals that received sitagliptin had higher cardiac mRNA and protein ACE2 expression as well as lower cardiac ACE/ACE2 ratios [132]. A systematic review and meta-analysis of nine studies evaluating DPP4 inhibitor use (both pre-hospital and in-hospital) and mortality in diabetic patients with COVID-19 infection were conducted. While this study found no data suggesting that DPP4 inhibitors may improve mortality [133] regardless of pre-hospital or in-hospital use, another study found that DPP4 inhibitors use in-hospital was associated with reduced mortality [134].

### 5.7. NSAIDs

Early in the pandemic, anecdotal reports of severe COVID-19 infection being associated with ibuprofen use raised concerns about the safety of NSAIDs use during infection. This resulted in advisories for the avoidance of NSAIDs if infected with SARS-CoV-2, favoring paracetamol as an alternative to alleviate COVID-19 infection symptoms [135]. Reports followed addressing the influence of NSAIDs on ACE2 expression and COVID-19 infection. In a healthy rat model, ibuprofen was found to increase the expression of ACE2 as well as anti-inflammatory receptors MasR and angiotensin type 2 (AT2R) while also decreasing the expression of proinflammatory receptor AT1R [136]. This same study also administered ibuprofen to a metabolic syndrome rat model and found ibuprofen reversed the proinflammatory RAS axis, shifting physiology to the more anti-inflammatory ACE2/ANG1–7/MasR axis. In vitro tissue cultures of human type II alveolar pneumocytes found upregulation of ACE2 expression in the presence of ibuprofen. In the presence of SARS-CoV-2 spike protein, ACE2 expression was attenuated without ibuprofen but similar to control cultures with the addition of ibuprofen [124]. A mouse model study designed to investigate the influence of ibuprofen, as well as flurbiprofen and etoricoxib, on ACE2 expression had contradictory findings. This group of scientists found no effect of these NSAIDs on ACE2 expression or activity [137].

## 6. Limitations

Limitations of this article include the broad inclusion and exclusion criteria for the literature search. Additionally, while we have reviewed a substantial number of articles on ACE2 expression in COVID-19 patients and drew limited conclusions to the best of our ability, as detailed above, a definitive answer on whether or not these differences affect COVID-19 infection susceptibility or severity has yet to be made in more extensive studies.

## 7. Summary

Given the distribution of ACE2 to many tissues throughout the body and the dependence of SARS-CoV-2 binding to ACE2 to infect human cells, understanding the interrelation of ACE2 and SARS-CoV-2 and the impact of ACE2 on human pathophysiology as it relates to patient characteristics and comorbidities is an important link to understanding the COVID-19 infection and its severity. In this literature review, we have collected insight from past publications regarding ACE2 and whether it may be differentially expressed due to patient characteristics, comorbidities, and medication use. In turn, we have also explored whether or not these differences correlate to disparities in infection susceptibility or disease severity.

Our impressions, based on our review, are that differences in ACE2 expression occur in differing patient characteristics, and these differences may be linked to worse severity of COVID-19 infection and poorer outcomes. An age-related increase in pulmonary expression of ACE2 in the pneumocytes of the lungs, as well as increased ACE2 expression in older patients who require ventilation in comparison to older non-ventilated patients, was associated with greater severity and mortality in COVID-19. Others have found opposing evidence, suggesting that downregulation of ACE2 expression is linked to the severity of ARDS and, therefore, proposing that higher ACE2 expression may be protective. Increases in acute kidney injury and mortality in COVID-19-infected patients may be linked to increased ACE2 expression in the same sites as of the renal tubular cells damaged by SARS-CoV-2. In contrast, the downregulation of ACE2 expression in patients with cardiovascular disease within cardiac myocytes with the unfavorable effect of an imbalance in the ACE/ACE2 ratio. These changes result in a decrease in the protective effects of the ACE2/ANG1–7/MasR axis and favor the detrimental effect of relatively underregulated ANG II activity and possibly leading to a higher incidence of myocardial infarction in appropriately predisposed patients. This mechanism of ACE2-SARS-CoV-2 interaction again worsened these patients’ outcomes but with a different mechanism. Moreover, medications associated with an increase in ACE2 expression, whether the circulating portion (metformin) or at the cardiovascular system (mineralocorticoid receptor antagonist, statins, GLP1-RA, DPP4i or NSAIDs), may provide protective effects by increasing ACE2 expression and improving outcomes from COVID-19 infection.

Based on this review, we may hypothesize that the difference in COVID-19 susceptibility and severity between two patients with similar age and comorbid profiles, which we have experienced clinically during the pandemic, can be related to the difference in ACE2 expression in their different organs.

Graphical Abstract Description: Modelling the ACE2/MasR and ACE/AT1R axis, and the factors associated with variable ACE2 expression. 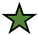 Factors with increased expression: Female sex, estrogen levels, younger age*, nasal and bronchial epithelial cells with age, mechanical ventilation with age, post-MI x 4 weeks, heart failure, renal proximal tubule cells, renal glomerular podocytes, pancreatic islet cells, controlled diabetes (1/2), Type 2 pneumocytes, COPD, chronic tobacco exposure, e-cigarettes, alcohol consumption, atopic asthma, ACEi, ARBs, MRAs, statins, GLP-1 receptor agonists, DPP4, NSAIDs*. 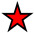 Factors with reduced expression: Male sex, older age*, SARS-CoV-2 infection of cardiac myocytes, hypertension, uncontrolled diabetes (1/2), glomerulus of CKD patients, se-vere ARDS*, NSAIDs*, *Inconsisent evidence.
